# Body mass, diabetes and smoking, and endometrial cancer risk: a follow-up study

**DOI:** 10.1038/sj.bjc.6604313

**Published:** 2008-03-25

**Authors:** K Lindemann, L J Vatten, M Ellstrøm-Engh, A Eskild

**Affiliations:** 1 Department of Obstetrics and Gynaecology, Medical Faculty, Division of Akershus University Hospital, 1478 Lørenskog, Norway; 2 Department of Public Health, Faculty of Medicine, Norwegian University of Science and Technology, 7289 Trondheim, Norway; 3 International Agency for Research on Cancer, 69372 Lyon, France; 4 Division of Mental Health, Norwegian Institute of Public Health, PO Box 4404 Nydalen, 0403 Oslo, Norway

**Keywords:** endometrial cancer, BMI, diabetes, smoking, alcohol, epidemiology

## Abstract

We examined the relationship of body mass index (BMI), diabetes and smoking to endometrial cancer risk in a cohort of 36 761 Norwegian women during 15.7 years of follow-up. In multivariable analyses of 222 incident cases of endometrial cancer, identified by linkage to the Norwegian Cancer Registry, there was a strong increase in risk with increasing BMI (*P*-trend <0.001). Compared to the reference (BMI 20–24 kg m^−2^), the adjusted relative risk (RR) was 0.53 (95% confidence interval (CI): 0.19–1.47) for BMI<20 kg m^−2^, 4.28 (95% CI: 2.58–7.09) for BMI of 35–39 kg m^−2^ and 6.36 (95% CI: 3.08–13.16) for BMI⩾40 kg m^−2^. Women with known diabetes at baseline were at three-fold higher risk (RR 3.13, 95% CI: 1.92–5.11) than those without diabetes; women who reported current smoking at baseline were at reduced risk compared to never smokers (RR 0.55, 95% CI: 0.35–0.86). The strong linear positive association of BMI with endometrial cancer risk and a strongly increased risk among women with diabetes suggest that any increase in body mass in the female population will increase endometrial cancer incidence.

Endometrial cancer is the most commonly diagnosed cancer of the female genital tract in developed countries (Parker *et al*, 1997). The increasing incidence is partly due to increased life expectancy, but changes in lifestyle factors are also likely to be important.

Previous studies have shown high body mass index (BMI) to be associated with increased risk of endometrial cancer (Tretli and Magnus, 1990; Swanson *et al*, 1993; Ballard-Barbash and Swanson, 1996; Goodman *et al*, 1997; Furberg and Thune, 2003; Jonsson *et al*, 2003; Schouten *et al*, 2006; Bjorge *et al*, 2007), and obesity has been estimated to account for about 40% of endometrial cancer incidence in affluent societies (Bergstrom *et al*, 2001). However, it is still not clear whether the effect of obesity on risk displays a linear relation, or if there is a threshold effect leading to higher risk only among obese women (BMI⩾30 kg m^−2^) (IARC, 2002).

Few studies have investigated a possible differential effect of BMI on endometrial cancer risk before and after menopause (La Vecchia *et al*, 1991; Tornberg and Carstensen, 1994). These studies tended to show a possible threshold effect in premenopausal women, whereas in older women there appears to be a linear increase with BMI. Diabetes has also been related to increased endometrial cancer risk (Goodman *et al*, 1997; Weiderpass *et al*, 1997; Shoff and Newcomb, 1998; Friberg *et al*, 2007), whereas several studies have shown a negative association with smoking (Austin *et al*, 1993; Brinton *et al*, 1993; Weiderpass and Baron, 2001; Viswanathan *et al*, 2005). These effects have been related to plasma concentrations of endogenous hormones, insulin and other growth factors.

The marked effect of obesity on endometrial cancer risk and the increasing body weight in many populations indicate that the incidence of this disease will continue to increase. To investigate this issue further, we have studied endometrial cancer risk in a prospective study of 36 761 women in relation to increments of BMI, diabetes and smoking in all women and separately in women younger than or older than 55 years of age.

## MATERIALS AND METHODS

During 1984–1986, a health survey was conducted among men and women aged ⩾20 years in Nord-Trøndelag county in Norway (the HUNT Study). Among 85 100 eligible persons, 77 310 (90.8%) returned the questionnaire that was mailed with the invitation (questionnaire 1), of whom 74 977 (38 274 women) participated in the subsequent clinical examination. At this clinical examination, standardised measurements of height, weight and blood pressure were performed, and participants received a second questionnaire that included items on physical activity, alcohol use, diabetes and smoking. This second questionnaire was filled in at home and returned in a prestamped envelope, but 6255 women (17%) did not do so. Details of the description of the HUNT Study are provided elsewhere (Holmen *et al*, 1991).

Among the 38 274 women who participated, 1094 were excluded because of prevalent cancer (of any site, except basal cell carcinoma) and 419 were excluded because BMI could not be calculated. Thus, 36 761 women constituted the study population and were followed up for cancer incidence.

The unique 11-digit identity number of Norwegian citizens enabled linkage of participants to endometrial cancer recorded at the Cancer Registry of Norway. All women so diagnosed among participants (International Classification of Diseases, 7th revision, ICD-7, code 172) were identified based on mandatory reporting from all pathological laboratories in Norway to the Cancer Registry.

Follow-up time was calculated as person-years from the date of clinical examination until the date of cancer diagnosis (except basal cell carcinoma), emigration, death or to the end of follow-up at 31 December 2002, whichever occurred first. In separate analyses among women before the age of 55 years, follow-up was the time from the date of clinical examination until endometrial cancer diagnosis or until censoring at the age of 55 years.

Body mass index was calculated as weight divided by height squared (kg m^−2^) and categorised as <20, 20–24, 25–29, 30–34, 35–39, and 40 kg m^−2^ and higher. At the clinical examination, blood pressure ⩾140/90 mmHg was classified as hypertension.

Smoking status was coded as never, former, current or ‘missing’. Alcohol drinking was categorised as 0, 1–4, ⩾5 times during the last 2 weeks, total abstainer or missing. Questions on recreational physical activity (i.e., walking, skiing, swimming or other sports) included frequency (five categories), average duration (four categories) and intensity (three categories). Marital status (married, unmarried, widow and divorced/separated) and level of education (<10, 10–12, >12 years or missing) were also recorded.

### Statistical analysis

We estimated the age-adjusted relative risks (RRs) of BMI, diabetes and smoking with 95% confidence intervals (CIs) by using the Cox regression analysis. Thereafter, we mutually adjusted for each study factor and also for alcohol use, physical activity and hypertension. We used the SPSS statistical package, version 14.0, for the analysis.

This study was approved by the Regional Committee for Ethics in Medical Research and the Norwegian Data Inspectorate.

## RESULTS

We followed 36 761 women for an average of 15.7 years (range 0–19 years). Mean age at baseline was 49 years (range 20–101 years), and during follow-up, 222 endometrial cancers were diagnosed.

Among women with BMI<20 kg m^−2^, only 0.13% (4 out of 3067) developed endometrial cancer compared to 3.66% (9 out of 246) of those with BMI⩾40 kg m^−2^. Only 0.7% women (246) were in the highest BMI category, but they represented 4.1% of those with endometrial cancer. We found a strong and consistent increase in risk with increasing BMI (*P*-trend <0.001) (Table 1). Compared to women with BMI of 20–24 kg m^−2^, the age-adjusted RR for BMI<20 kg m^−2^ was 0.51 (95% CI: 0.19–1.40), and with BMI⩾40 kg m^−2^, it was 7.89 (95% CI: 3.90–15.94). Adjusting for diabetes, smoking status, alcohol use, physical activity and hypertension reduced the associations with BMI, but they remained strong. The adjusted RRs were 6.36 (95% CI: 3.08–13.16) for BMI⩾40 kg m^−2^, 4.28 (95% CI: 2.58–7.09) for BMI 35–39 kg m^−2^ and 0.53 (95% CI: 0.19–1.47) for BMI<20 kg m^−2^, respectively (Table 1).

Among women with diabetes at baseline, 1.88% women (19 out of 1010) were diagnosed with endometrial cancer during follow-up compared to 0.57% (203 out of 35 751) among women without diabetes. After multivariable adjustment, diabetes was associated with a three-fold higher risk (RR 3.13, 95% CI: 1.92–5.11) (Table 1).

There was an inverse association of current smoking with risk. After multivariable adjustment, the negative association with smoking was moderately reduced but remained strong (RR 0.55, 95% CI: 0.35–0.86); former smoking was not associated with risk (RR 1.06, 95% CI: 0.71–1.61) (Table 1).

Because information on age at menopause was not available, we conducted separate analyses before and after the age of 55 years. Among the 22 027 women who could be followed until the age of 55 years, 52 developed endometrial cancer, but multivariate analyses revealed a significantly increased risk only for women with BMI⩾35 kg m^−2^. The RRs were 6.10 (95% CI: 1.95–19.05) for BMI 35–39 kg m^−2^ and 9.44 (95% CI: 2.01–44.38) for BMI⩾40 kg m^−2^ (Table 2) as compared to the reference group (BMI 20–24 kg m^−2^).

Among women older than 55 years, 170 cases were diagnosed during follow-up. These showed a linear increase with increasing BMI, and RRs were 6.07 (95% CI: 2.65–13.93) associated with BMI⩾40 kg m^−2^ and 4.20 (95% CI: 2.37–7.47) with BMI 35–39 kg m^−2^ (Table 2).

Initially, marital status and educational level were included, but since they were not associated with risk, they were not included in the final analysis.

## DISCUSSION

Among the 36 761 women followed for 15.7 years, we found a strong positive and linear association of BMI with endometrial cancer risk in the study population as a whole. We found a six-fold increase in risk among very obese women (⩾40 kg m^−2^) compared to those of normal BMI (20–24 kg m^−2^), whereas those women with BMI<20 kg m^−2^ had only half the risk. Women with diabetes had three-fold higher risk compared to non-diabetic women, and we found an inverse association with smoking at baseline.

A weakness of our study is the lack of control for reproductive factors, such as parity, oral contraceptive use, hormone replacement therapy (HRT) and possible changes in BMI during follow-up. Generally, Norwegian women were restrictive in HRT use in the 1980s, with an estimated less than 6% of postmenopausal women using it in the late 1980s (Graff-Iversen *et al*, 1998). Hormone replacement therapy use increased in the 1990s when about 35% of postmenopausal women reported using it. However, users of combined oestrogen–progesterone preparations, who constituted 70% of all Norwegian users, have had no increase in endometrial cancer risk (Bakken *et al*, 2004). Women who had undergone hysterectomy could not be excluded in our study because of the lack of such information. The hysterectomy rate due to benign disease has been low in Norway, although it has increased in recent years (Kalseth and Backe, 2002).

Even though the association between obesity and endometrial cancer is ‘convincing’ (IARC, 2002), it is still not clear if this displays a linear gradient. Besides a linear relation, with no evidence for any threshold effect in the population as a whole, the associations in our study were stronger than in most previous studies (Furberg and Thune, 2003; Jonsson *et al*, 2003; Schouten *et al*, 2006). The higher impact of BMI on risk may be because previous studies did not address the effect of very high body mass separately, while others did not adjust for potentially confounding factors (Tretli and Magnus, 1990; Bjorge *et al*, 2007).

The association with diabetes is also stronger than that previously reported (Goodman *et al*, 1997; Weiderpass *et al*, 1997; Shoff and Newcomb, 1998; Friberg *et al*, 2007). In line with most studies (Austin *et al*, 1993; Brinton *et al*, 1993; Weiderpass and Baron, 2001; Viswanathan *et al*, 2005), we found an inverse association with smoking at baseline after controlling for potential confounding by other factors.

The fact that obesity increases risk has been attributed to changes in concentrations of endogenous hormones in obese women. Oestrogens produced in adipose tissue have a direct mitogenic effect on endometrial cells, and in obese women, this effect is assumed not to be counterbalanced by progesterone because of chronic anovulation and thereby much reduced progesterone synthesis. It has even been argued that low progesterone, rather than increased oestrogens, is the predominant determinant of endometrial cancer in premenopausal women and that the increased risk is only related to oestrogens when oestrogen concentrations are comparatively low, as found in postmenopausal women.

Progesterone counterbalances oestrogen and diminishes oestrogenic action in the endometrium. As obesity is related to both anovulation and low progesterone, these mechanisms are irrelevant in younger women with high BMI. In postmenopausal women, oestrogens derived from peripheral adipose tissue are the primary source of endogenous E_2_, and the rate of production is related to the size of the adipose depots. It can be argued that only in those women, with comparably low oestrogen concentration, risk is directly related to circulating oestrogen. Our results may support the hypothesis of different mechanisms in different age groups leading to a threshold effect of BMI in younger premenopausal women. However, data from this study population are insufficient to draw a definite conclusion.

There are indeed several possible mechanisms related to body mass that are identical in pre- and postmenopausal women. Firstly, there is a weight-related increase in insulin and insulin-like growth factor-I (IGF-I), both of which are endometrial growth factors (Crave *et al*, 1995; Pasquali *et al*, 1997). Secondly, cytokines produced in fat tissue (leptin and adiponectin) may play a direct role in endometrial carcinogenesis (Petridou *et al*, 2002; Dal Maso *et al*, 2004; Housa *et al*, 2006), as well as transcription factors that can modulate both cellular lipid metabolism and tumorigenesis (Roberts-Thomson, 2000; Fajas *et al*, 2001).

Apart from the effects of unopposed oestrogens, insulin and associated growth factors have been identified as risk factors. We found a positive association of diabetes with risk, also after adjusting for potentially confounding factors. Several mechanisms may operate in linking elevated insulin to endometrial cancer development, including growth-enhancing properties of insulin, increased levels of IGF-I receptors in the cancer tissue (Talavera *et al*, 1990; Roy *et al*, 1999) and suppressed gene expression of endometrial IGFBP-1, leading to increased biological activity of IGF-I (Irwin *et al*, 1993; Ayabe *et al*, 1997).

The protective effect of smoking on risk cannot be entirely attributed to lower body weight. Reversible processes are probably involved, since the finding was restricted to current, but not former, smokers. Smoking may slow down the decay of progesterone and androgens (Sowers *et al*, 2001), and thereby reduce oestrogen-mediated cellular proliferation and mutations in endometrial glands. It also has a direct anti-oestrogenic effect, and there appears to be a direct toxic effect on the ovaries (Matikainen *et al*, 2001).

In this prospective study of a large unselected population, the linear and strong positive association of BMI with risk implies that even a slight increase in population body mass will lead to an increase in endometrial cancer incidence. More detailed research is indicated.

## Figures and Tables

**Table 1:** BMI, diabetes and smoking, and RR of endometrial cancer in the study population of 36 761 women in Norway

**Variable**	**No. of persons**	**No. of cases**	**Age-adjusted RR^a^**	**Multivariate RR^b^**	**95% CI**	***P* for trend**
*BMI (kg m*^*−2*^)						
<20	3067	4	0.51	0.53	0.19–1.47	
⩾20–24	17 966	64	1.0	1.0		
⩾25–29	10 830	90	1.83	1.74	1.25–2.43	
⩾30–34	3680	32	1.88	1.66	1.06–2.59	
⩾35–39	972	23	5.04	4.28	2.58–7.09	
⩾40	246	9	7.89	6.36	3.08–13.16	<0.001
						
*Diabetes*						
No	35 751	203	1.0	1.0		
Yes	1010	19	3.84	3.13	1.92–5.11	<0.001
						
*Smoking*						
Never	16 433	122	1.0	1.0		
Former	4635	31	1.07	1.06	0.71–1.61	
Current	9438	26	0.49	0.55	0.35–0.86	
Missing	6255	43	1.14	1.05	0.56–1.97	0.09

BMI=body mass index; CI=confidence interval; RR=relative risk.

a Adjusted for age in 10-year categories (<30, 30–39, 40–49, 50–59, 60–69 and ⩾70 years).

b Adjusted for age in 10-year categories (<30, 30–39, 40–49, 50–59, 60–69 and ⩾70 years), physical activity (no, low, moderate, high and missing), hypertension (<140/90 and ⩾140/90 mmHg) and alcohol consumption during the last 2 weeks (abstinent, never, 1–4 times, >5 times and missing) and each study factor.

**Table 2:** BMI and RR of endometrial cancer in women younger and older than 55 years of age

**Age (years)**	**BMI (kg m^−2^)**	**No. of persons**	**No. of cases**	**Age-adjusted RR^a^**	**Multivariate RR^b^**	**95% CI**	***P* for trend**
<55	<20	2515	3	0.7	0.75	0.23–2.50	
	⩾20–24	13 030	25	1.0	1.0		
	⩾25–29	4851	15	1.56	1.49	0.78–2.87	
	⩾30–34	1206	3	1.27	1.24	0.37–4.20	
	⩾35–39	333	4	6.13	6.10	1.95–19.05	
	⩾40	92	2	10.18	9.44	2.01–44.38	0.007
							
⩾55	<20	1205	1	0.27	0.28	0.04–2.01	
	⩾20–24	10 485	39	1.0	1.0		
	⩾25–29	8906	75	1.95	1.85	1.25–2.75	
	⩾30–34	3224	29	2.06	1.77	1.07–2.93	
	⩾35–39	868	19	5.04	4.20	2.37–7.47	
	⩾40	220	7	7.79	6.07	2.65–13.93	<0.001

BMI=body mass index; CI=confidence interval; RR=relative risk.

a Adjusted for age in 10-year categories (<30, 30–39, 40–49, 50–59, 60–69 and ⩾70 years).

b Adjusted for age in 10-year categories (<30, 30–39, 40–49, 50–59, 60–69 and ⩾70 years), diabetes (yes/no), smoking (never, former, current and missing), physical activity (no, low, moderate, high and missing), hypertension (<140/90 and ⩾140/90 mmHg) and alcohol consumption during the last 2 weeks (abstinent, never, 1–4 times, >5 times and missing).
